# Supplementary material for: Elevated binding and functional antibody responses to SARS-CoV-2 in infants versus mothers
Source: Nat Commun. 2023 Aug 11;14:4864. doi: 10.1038/s41467-023-40554-w (PMC10421871; doi:10.1038/s41467-023-40554-w)
Supplement: Supplementary file 1 — Supplementary Information [file 41467_2023_40554_MOESM1_ESM.pdf]

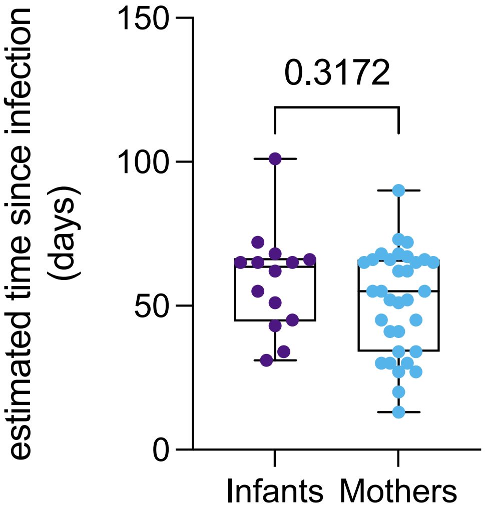

Figure S1. **Estimated time since infection for infants versus mothers.** Time since infection was estimated by calculating the midpoint between the last seronegative sample and the first seronegative sample among infants (N = 14) and mothers (N = 33) (see Methods). Two-tailed Wilcoxon rank sum test was used for comparison. P-value is indicated above the comparison. Box plots show median center line and 25/75 percentiles. Whiskers show min and max values.

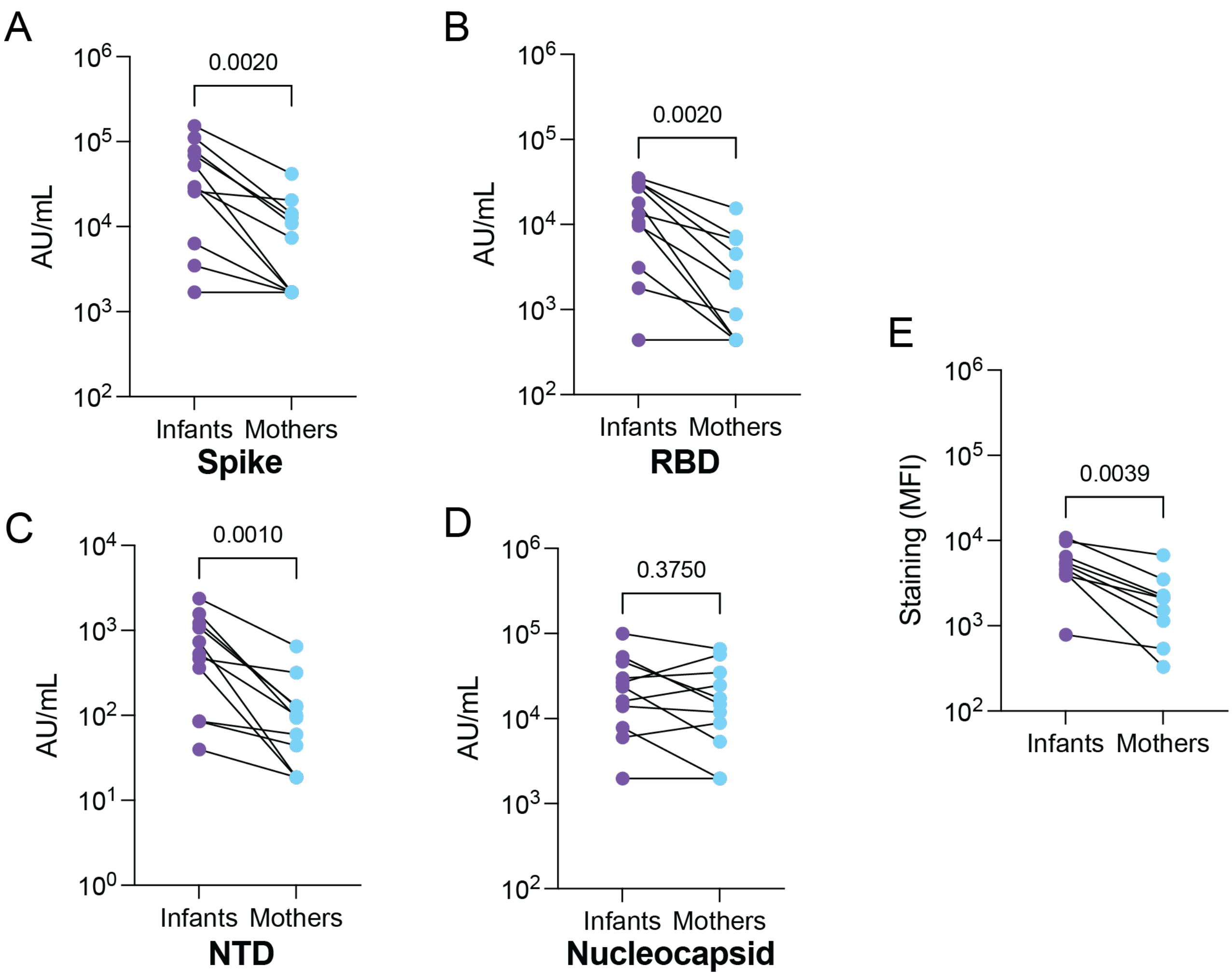

Figure S2. **Infant-mother paired analyses of SARS-CoV-2 antigen binding and relationship between MSD and cell-surface staining measurements.** IgG antibody titers among infant-mother pairs (N = 11) for SARS-CoV-2 Spike (A), RBD (B), NTD (C), and Nucleocapsid (D) measured by multiplexed MSD immunoassay. (E) S-CEM cells surface staining with plasma among infant-mother pairs (N = 9). MFI: mean fluorescence intensity. Two-tailed Wilcoxon matched pairs sign-rank test was used for paired comparisons. P-values and Spearman correlation coefficient are indicated above all comparisons.

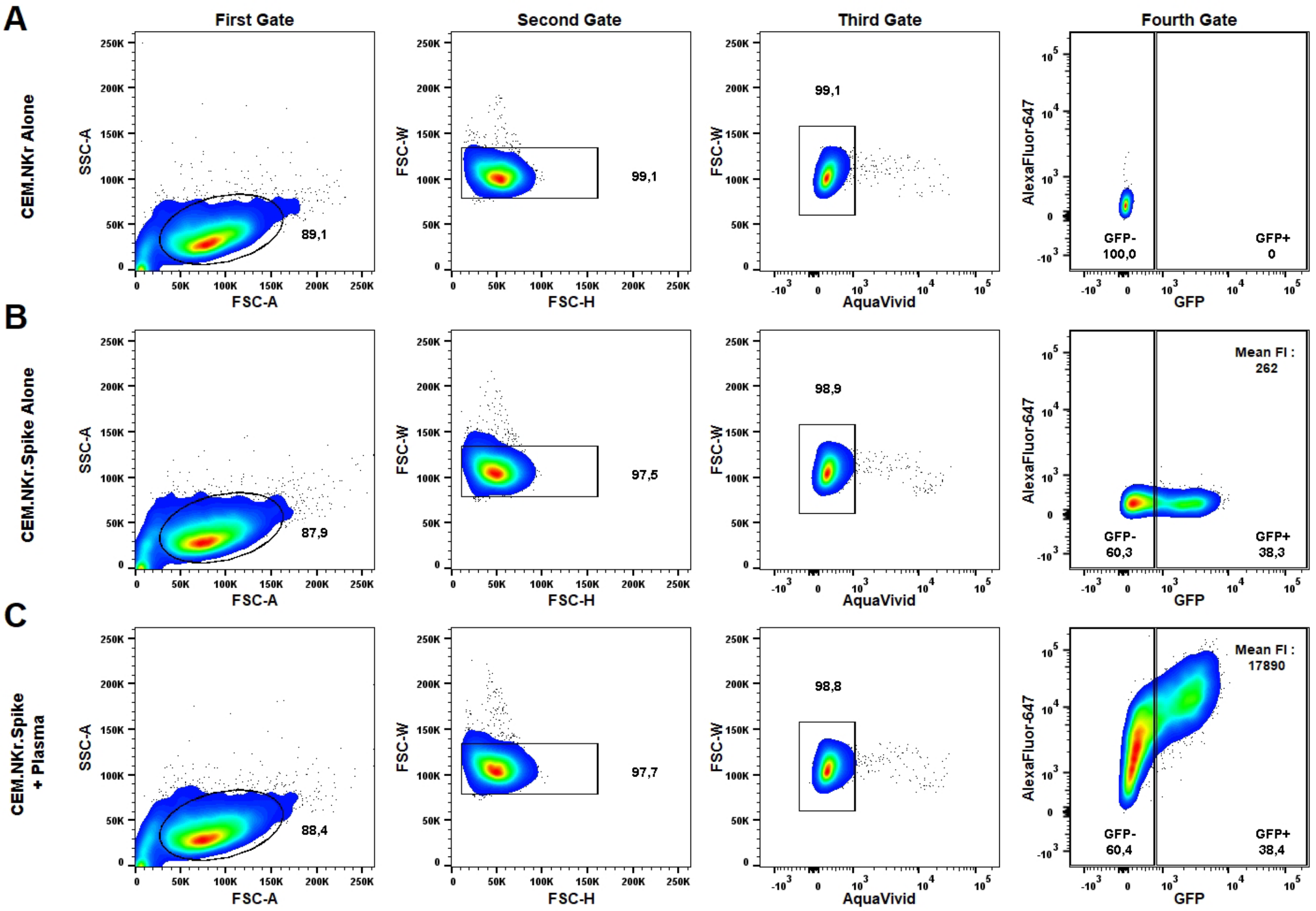

Figure S3. **Gating strategy for cell surface staining measurements.** Cells were identified according to cell morphology by light-scatter parameters (first column) and excluding doublets cells (second column). Dead cells ($Aqua^{High}$) were then excluded (third column). Finally, the GFP+ cells were used to detect and measure the Spike-specific antibodies present in the plasma (fourth column). Examples of typical gating using (A) parental CEM.NKr or (B) CEM.NKr.Spike cells in absence or in (C) presence of plasma.

	Unstratified mothers vs. infants (Figs. 1, 2, 5)			HEU infants vs. MLWH			HUU infants vs. HIV-uninfected mothers		
	Median (inf, mom)	p-value (inf, mom)	N (inf, mom)	Median (inf, mom)	p-value (inf, mom)	N (inf, mom)	Median (inf, mom)	p-value (inf, mom)	N (inf, mom)
**MSD Spike**	34808, 6622	0.002	14, 36	34808, 8142	0.006	8, 20	39607, 1686	0.02	6, 16
**MSD RBD**	15580, 2247	0.001	14, 36	14513, 2574	0.006	8, 20	15580, 438.8	0.02	6, 16
**MSD NTD**	561.9, 91.5	0.001	14, 36	561.9, 110.6	0.005	8, 20	398.3, 18.7	0.01	6, 16
**MSD nucleocapsid**	21945, 13311	1.0	14, 36	23418, 22621	0.9	8, 20	18869, 5261	0.3	6, 16
**S-CEM staining**	5332, 2111	0.0009	10, 35	5551, 1835	0.03	6, 20	5332, 2291	0.02	4, 14
**FP summed enrichment**	274.3, 145.1	0.01	10, 35	274.3, 139.3	0.03	6, 20	260.5, 146.6	0.4[+]	4, 15
**SH-H summed enrichment**	91.6, 88.2	0.8	10,35	80.5, 97.7	0.5	6, 20	117.2, 78.8	0.2	4, 15
**ADCC activity**	35.1, 19.7	0.0002	10, 35	31.8, 19.5	0.03	6, 20	36.3, 21.3	0.003	4, 14

Table S1. **Influence of HIV status stratification on comparisons of antibody binding and activity between infants and their mothers**. P-values are indicated for comparisons of infants and their mothers in aggregate or stratified by HIV status for the indicated assays. Two-tailed Wilcoxon rank sum test was used for all comparisons. Directionality of significant comparisons did not change for all stratified comparisons. Original figures are provided for unstratified p-values, for reference. [+]Indicates p-value that was no longer significant after stratification. MLWH: mothers living with HIV; HEU: HIV exposed uninfected infants; HUU: HIV unexposed uninfected infants.

	Unstratified mothers vs. infants (Figs. 1, 2, 5)			Asymptomatic infants vs. mothers			Mild symptom(s) infants vs. mothers		
	Median (inf, mom)	p-value (inf, mom)	N (inf, mom)	Median (inf, mom)	p-value (inf, mom)	N (inf, mom)	Median (inf, mom)	p-value (inf, mom)	N (inf, mom)
**MSD Spike**	34808, 6622	0.002	14, 36	40179, 4087	0.004	11, 28	29438, 14244	1.0*	3, 8
**MSD RBD**	15580, 2247	0.001	14, 36	17900, 1821	0.003	11, 28	10476, 5393	1.0*	3, 8
**MSD NTD**	561.9, 91.5	0.001	14, 36	594.5, 74.4	0.004	11, 28	361.4, 239.8	1.0*	3, 8
**MSD nucleocapsid**	21945, 13311	1.0	14, 36	23732, 8726	1.0	11, 28	16008, 45164	1.0	3, 8
**S-CEM staining**	5332, 2111	0.0009	10, 35	5332, 2006	0.002	8, 29	7496, 3168	0.4*	2, 6
**FP summed enrichment**	274.3, 145.1	0.01	10, 35	217, 144.3	0.05	8, 29	522, 180.7	0.3*	2, 6
**SH-H summed enrichment**	91.6, 88.2	0.8	10,35	84.2, 89.3	0.9	8, 29	97.2, 75.4	0.4	2, 6
**ADCC activity**	35.1, 19.7	0.0002	10, 35	(34.8, 19.2)	0.003	(7, 28)	(37.8, 23.4)	0.1*	(3,7)

Table S2. **Influence of symptom status stratification on comparisons of antibody binding and activity between infants and their mothers**. P-values, medians and N are indicated for comparisons of infants and their mothers in aggregate or stratified by symptom status for the indicated assays. Two-tailed Wilcoxon rank sum test was used for all comparisons. Directionality of significant comparisons did not change for all stratified comparisons. Original data (Unstratified mothers versus infants) are provided for unstratified p-values, for reference. *Indicates p-value that was no longer significant after stratification.

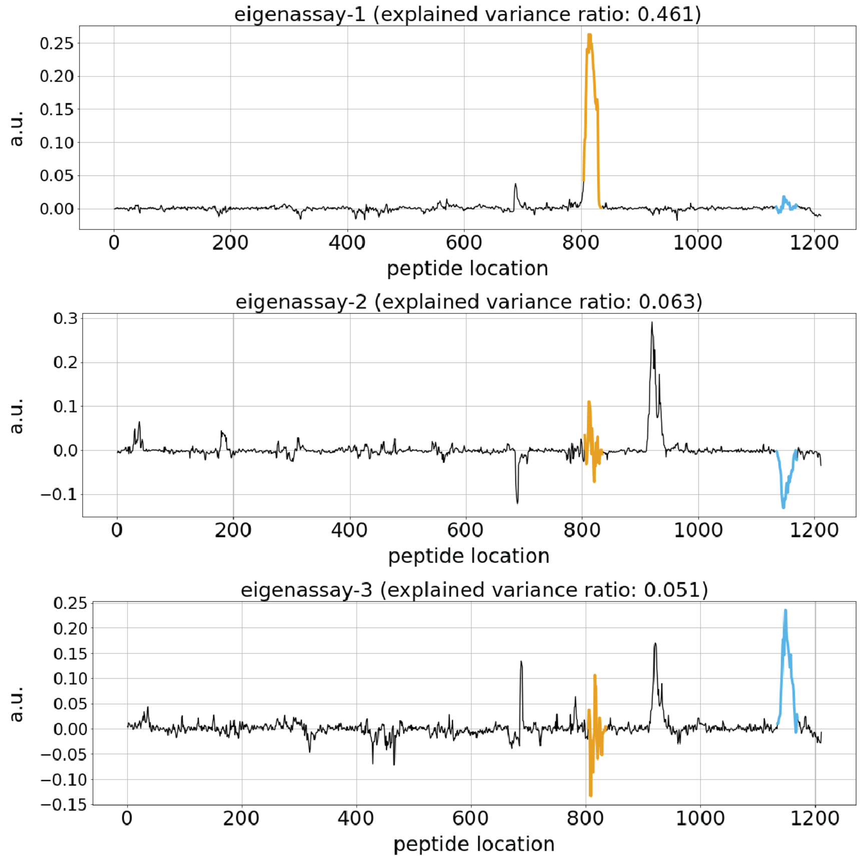

Figure S4. **Principal component analysis (PCA) to identify features of antibody enrichment at wildtype S sequences.** Line plots depicting the first three principal components from PCA analysis on the enrichment profiles from all infants and mothers. Regions showing large deviation from zero suggest high variation. The yellow and blue highlighted segments correspond to the FP and SH-H regions, respectively. The peak between positions 900-1000 in the latter two principal components was due to a single individual showing strong enrichment in that region and thus that region was not analyzed further.

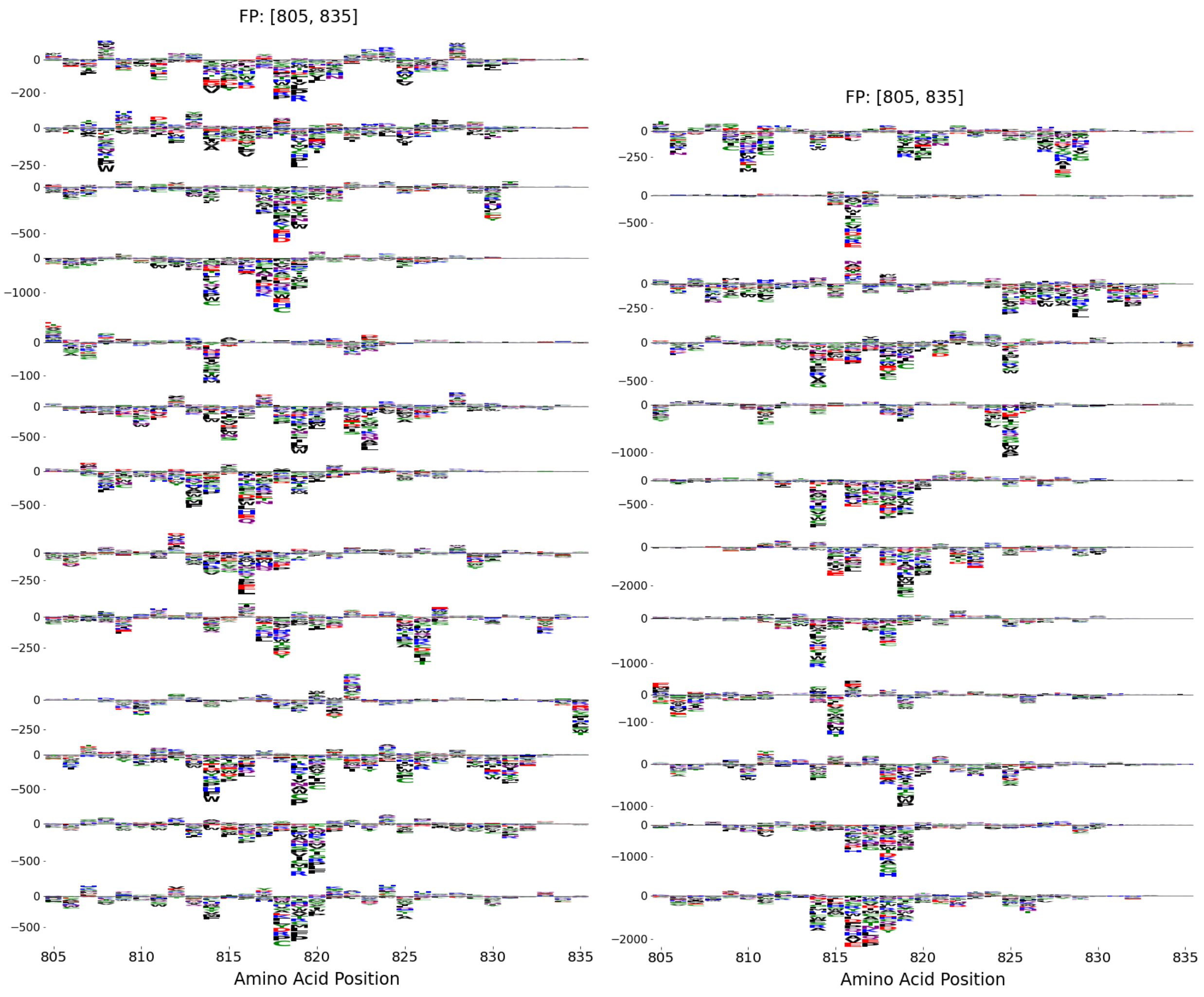

Figure S5. **Additional mutational escape profiles from mothers in FP region.** Logo plots depicting antibody escape profiles across the FP region in remaining mothers not shown in **Fig. 3B**.

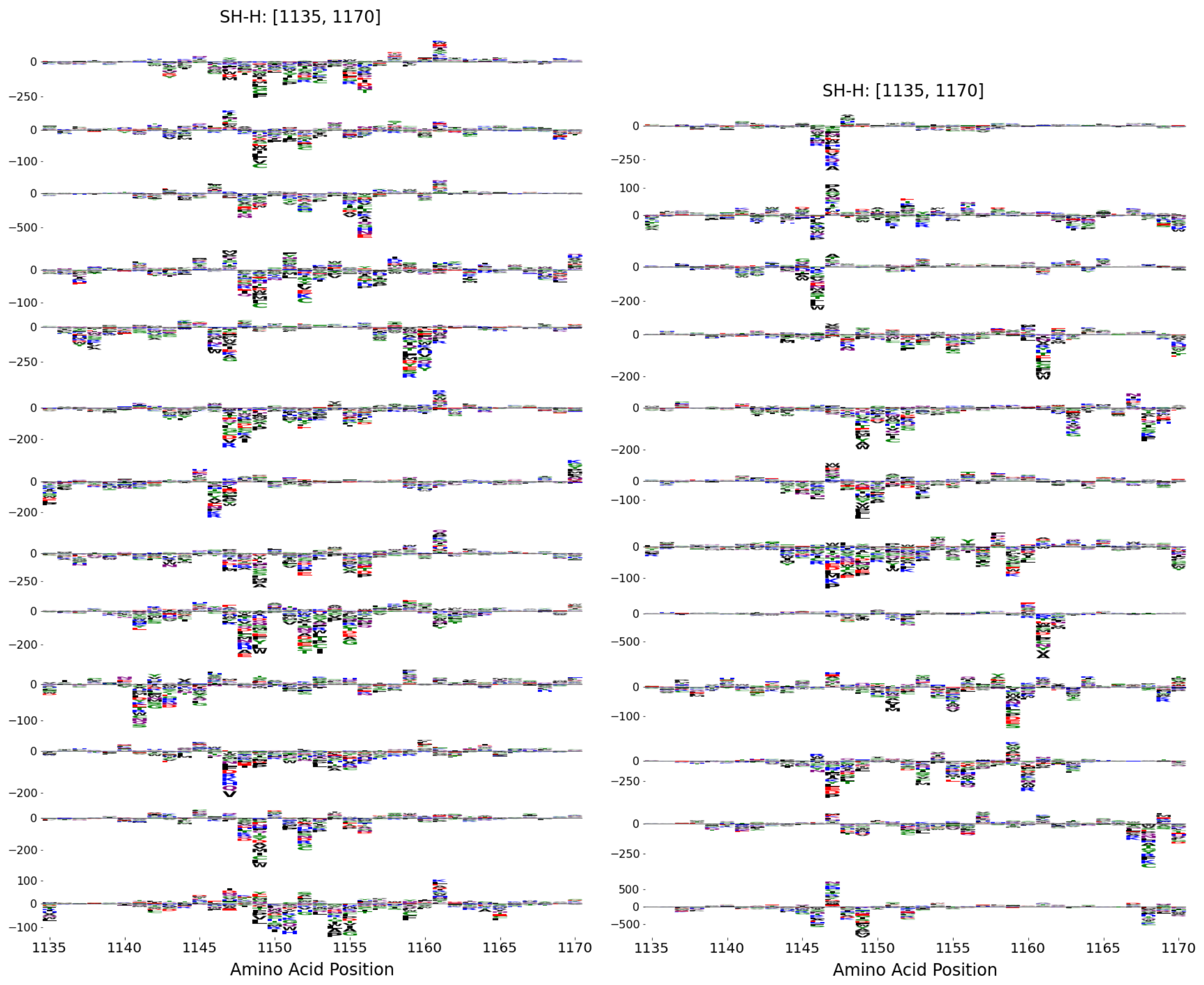

Figure S6. **Additional mutational escape profiles from mothers in SH-H region.** Logo plots depicting antibody escape profiles across the SH-H region in remaining mothers not shown in **Fig. 4B**.

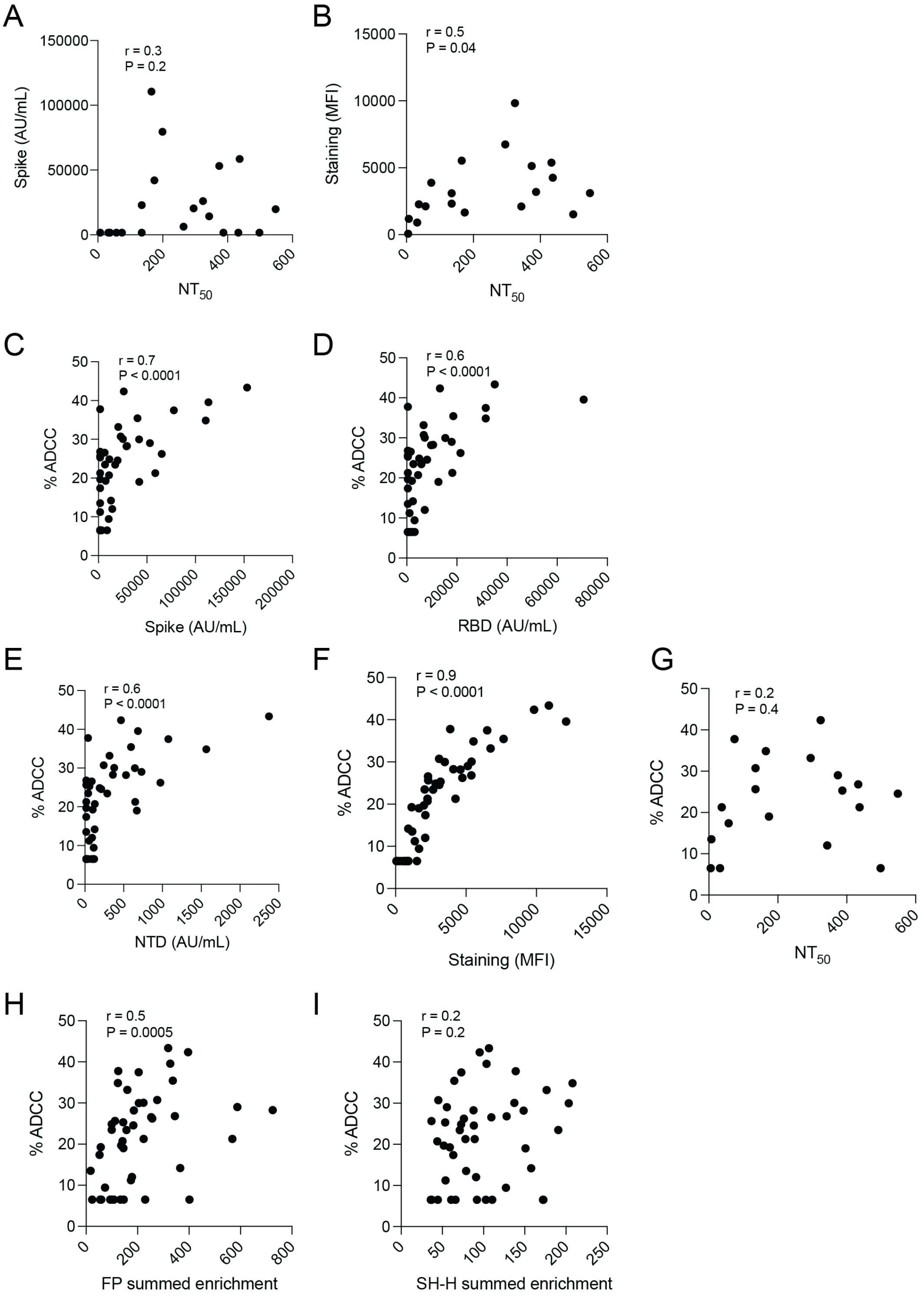

Figure S7. **Relationships between plasma antibody binding and functional activity assays for all infants and mothers.** (A) Spearman correlation between neutralization activity and Spike IgG binding by MSD assay. (B) Spearman correlation between neutralization data and S-CEM cell surface staining. (C) Spearman correlation between Spike IgG binding by MSD assay and ADCC activity ($p = 1.8x10^{-6}$). (D) Spearman correlation between RBD IgG binding by MSD assay and ADCC activity ($p = 7.9x10^{-5}$). (E) Spearman correlation between NTD IgG binding by MSD assay and ADCC activity ($p = 3.7x10^{-6}$). (F) Spearman correlation between S-CEM cell surface staining and ADCC activity. (G) Spearman correlation between neutralization activity and ADCC activity. (H) Spearman correlation between FP summed enrichment and ADCC activity. (I) Spearman correlation between SH-H summed enrichment and ADCC activity. P-values and Spearman correlation coefficients are indicated for each comparison. All Spearman tests were two-tailed. Comparisons include all available infants and mothers for each assay.

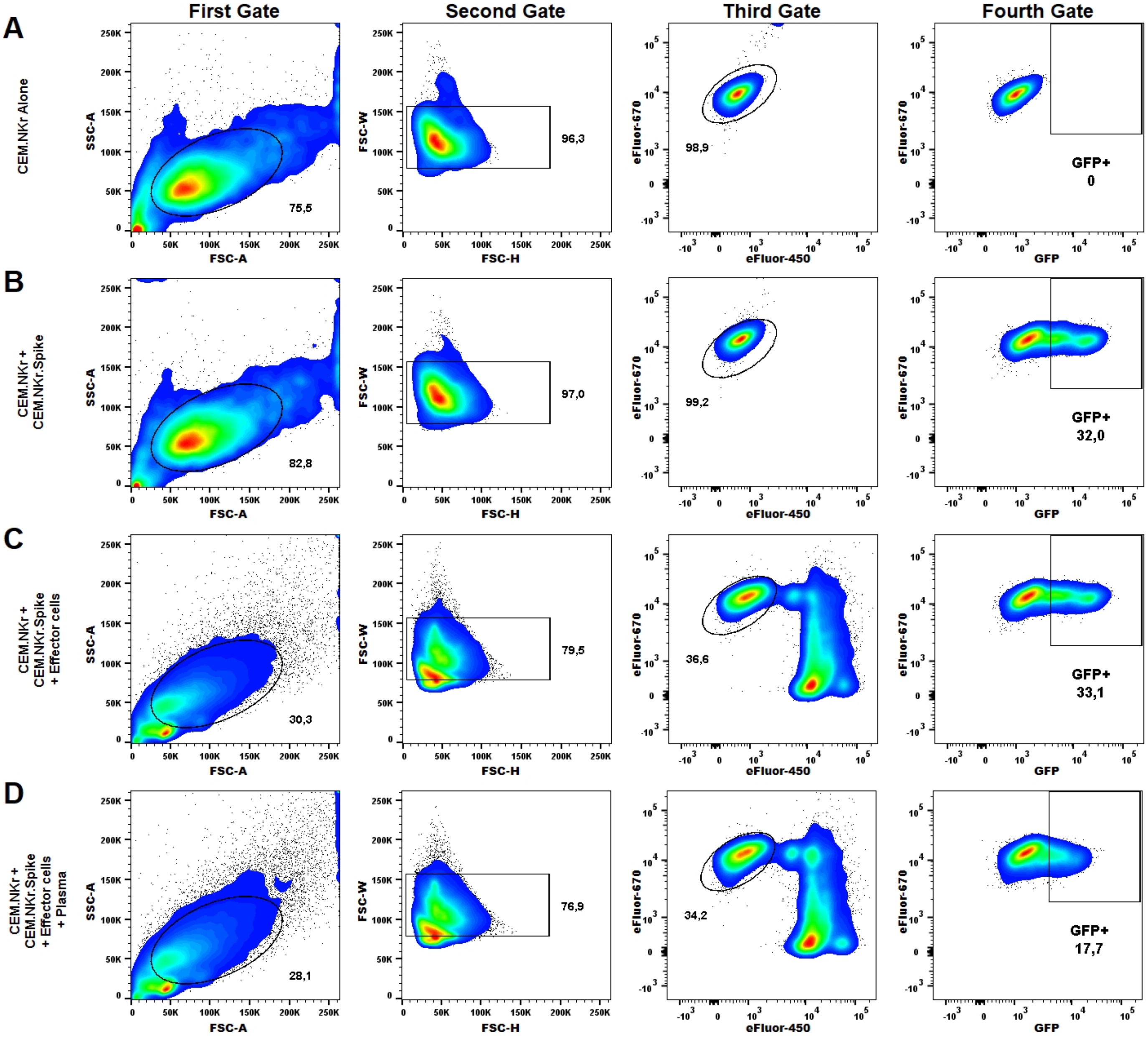

Figure S8. **Gating strategy for ADCC measurements.** Target cells were identified according to cell morphology by light-scatter parameters (first column) and excluding doublets cells (second column). Cells were then gated on eFluor-670+ cells (excluding the effector cells labeled with eFluor-450; third column). Finally, the percentage of GFP+ target cells was used to calculate the ADCC activity (fourth column). Examples of gating using (A) parental CEM.NKr or a (B) mix of parental CEM.NKr and CEM.NKr.Spike as target cells in absence or in (C) presence of effector cells to which was added a (D) plasma.
