# Supplementary material for: Elevated binding and functional antibody responses to SARS-CoV-2 in infants versus mothers
Source: Nat Commun. 2023 Aug 11;14:4864. doi: 10.1038/s41467-023-40554-w (PMC10421871; doi:10.1038/s41467-023-40554-w)
Supplement: Supplementary file 3 — Reporting Summary [file 41467_2023_40554_MOESM3_ESM.pdf]

nature portfolio

Corresponding author(s): Julie Overbaugh

Last updated by author(s): Jun 23, 2023

Reporting Summary

Nature Portfolio wishes to improve the reproducibility of the work that we publish. This form provides structure for consistency and transparency in reporting. For further information on Nature Portfolio policies, see our Editorial Policies and the Editorial Policy Checklist.

Statistics

For all statistical analyses, confirm that the following items are present in the figure legend, table legend, main text, or Methods section.

n/a	Confirmed	
☐	☒	The exact sample size (*n*) for each experimental group/condition, given as a discrete number and unit of measurement
☐	☒	A statement on whether measurements were taken from distinct samples or whether the same sample was measured repeatedly
☐	☒	The statistical test(s) used AND whether they are one- or two-sided<br>*Only common tests should be described solely by name; describe more complex techniques in the Methods section.*
☒	☐	A description of all covariates tested
☐	☒	A description of any assumptions or corrections, such as tests of normality and adjustment for multiple comparisons
☒	☐	A full description of the statistical parameters including central tendency (e.g. means) or other basic estimates (e.g. regression coefficient) AND variation (e.g. standard deviation) or associated estimates of uncertainty (e.g. confidence intervals)
☐	☒	For null hypothesis testing, the test statistic (e.g. *F*, *t*, *r*) with confidence intervals, effect sizes, degrees of freedom and *P* value noted<br>*Give P values as exact values whenever suitable.*
☒	☐	For Bayesian analysis, information on the choice of priors and Markov chain Monte Carlo settings
☒	☐	For hierarchical and complex designs, identification of the appropriate level for tests and full reporting of outcomes
☐	☒	Estimates of effect sizes (e.g. Cohen's *d*, Pearson's *r*), indicating how they were calculated

Our web collection on statistics for biologists contains articles on many of the points above.

Software and code

Policy information about availability of computer code

Data collection	Data using the Mesoscale Discovery (MSD) multiplexed immunoassay pipeline were collected on their proprietary plate reader using commercial Methodical Mind and Workbench (version 4.0) software. Flow cytometry data were collected with built-in BD FACSDiva software (v6). Spectroscopic data for pseudovirus assays were collected using built-in Omega software (v5.50 R4).
Data analysis	Phage-DMS data and escape profile similarity scores were analyzed using custom, publicly available pipelines built with python (v3.6.12), bowtie2 (v2.4.2), samtools (v1.3), and xarray (v0.16.1) software packages. Code and documentation can be found at https://matsengrp.github.io/phippery/ and https://matsengrp.github.io/phippery/esc-prof.html, respectively. Additional statistical analyses including Wilcoxon rank sum tests, and Wilcoxon matched-pairs signed rank tests and Spearman correlation were performed in Prism software (v9, Graphpad). Flow cytometry data were analyzed using FlowJo software (v10.7.1, TreeStar).

For manuscripts utilizing custom algorithms or software that are central to the research but not yet described in published literature, software must be made available to editors and reviewers. We strongly encourage code deposition in a community repository (e.g. GitHub). See the Nature Portfolio guidelines for submitting code & software for further information.

Data

Policy information about availability of data

All manuscripts must include a data availability statement. This statement should provide the following information, where applicable:

- Accession codes, unique identifiers, or web links for publicly available datasets
- A description of any restrictions on data availability
- For clinical datasets or third party data, please ensure that the statement adheres to our policy

Unprocessed sequencing data can be found at SRA accession PRJNA872509. The antibody binding, cell-surface staining, neutralization, and ADCC data generated in this study are provided in the Supplementary Information/Source Data file.

Human research participants

Policy information about studies involving human research participants and Sex and Gender in Research.

Reporting on sex and gender	The study cohort included mother (female sex) and their infants, though this study did not include analyses depending on infant sex, and this information is not included in the manuscript.
Population characteristics	Participant age is included in the manuscript for reference only, and was not considered quantitatively in the analyses. HIV status of mothers and infants was the only other demographic parameter included in the study, as data were stratified based on this information to confirm HIV status was not a deterministic driver of significant comparisons. This information is contained in Tables 1 and S1 of the manuscript.
Recruitment	Samples were included in the sub-study under the umbrella of the Linda Kizazi study based in Nairobi, Kenya. Pregnant women in their third trimester were recruited from Mathare North Health Centre in Nairobi. Women were eligible if aged 18–40 years, between 28–42 weeks gestation, planning to breastfeed, and, if living with HIV, had received ≥6 months of ART. Exclusion criteria included planned Caesarean section, serious medical condition, and taking antimicrobial or immunosuppressive medication other than for HIV prophylaxis (recruitment parameters quoted from: Begnal et al., PLoS One, 2022).
Ethics oversight	The Kenyatta National Hospital-University of Nairobi Ethics and Research Committee, the CHUM Research Center, and the University of Washington and Fred Hutchinson Cancer Center Institutional Review Boards approved of all Human Subjects study procedures.

Note that full information on the approval of the study protocol must also be provided in the manuscript.

Field-specific reporting

Please select the one below that is the best fit for your research. If you are not sure, read the appropriate sections before making your selection.

☒ Life sciences ☐ Behavioural & social sciences ☐ Ecological, evolutionary & environmental sciences

For a reference copy of the document with all sections, see nature.com/documents/nr-reporting-summary-flat.pdf

Life sciences study design

All studies must disclose on these points even when the disclosure is negative.

Sample size	Sample size was dictated by the number of Linda Kizazi study participants that seroconverted to SARS-CoV-2 during the sub-study period. Sample size was additionally limited by the exclusion of some seropositive infant samples due to low plasma sample volume. Collecting high volumes of plasma from infants is challenging and cumbersome for the infant and their family, so care was taken to reduce volumes when possible, thus sometimes limiting our infant sample size.
Data exclusions	There were no data exclusions.
Replication	Neutralization, phage-DMS, cell surface staining and ADCC experiments were conducted in technical singlicate or duplicate and biological duplicate or triplicate to ensure reproducibility. All attempts at replication were successful for these experiments. Because of limited plasma supply, especially from infant samples, MSD immunoassay measurements were conducted in singlicate, though efforts were made to confirm binding analyses using a parallel method, Spike surface staining, to compare with Spike binding measured by MSD. Those data were highly correlated, as indicated in the manuscript.
Randomization	Samples were allocated randomly into groups.
Blinding	Investigators were blinded to sample group during all experiments by assigning and randomly ordering sample IDs.

Reporting for specific materials, systems and methods

We require information from authors about some types of materials, experimental systems and methods used in many studies. Here, indicate whether each material, system or method listed is relevant to your study. If you are not sure if a list item applies to your research, read the appropriate section before selecting a response.

Materials & experimental systems

n/a	Involved in the study
☐	☒ Antibodies
☐	☒ Eukaryotic cell lines
☒	☐ Palaeontology and archaeology
☒	☐ Animals and other organisms
☒	☐ Clinical data
☒	☐ Dual use research of concern

Methods

n/a	Involved in the study
☒	☐ ChIP-seq
☐	☒ Flow cytometry
☒	☐ MRI-based neuroimaging

Antibodies

Antibodies used	Antibodies used as positive controls in ADCC experiments: CR3022 (Abcam), CV3-1, CV3-13, CV3-25, CV3-25 GASDALIE (CV3 antibodies provided by Andres Finzi lab)<br>ADCC negative control antibody: HIV-specific monoclonal antibody 17b (produced in Overbaugh lab using available sequence)<br>Secondary used for cell surface staining: Goat anti-human IgG (H+L) Alexa647 secondary antibody (Invitrogen, Catalog # A-21445)
Validation	Human monoclonal CR3022 antibody (Abcam, cat# ab278112) was validated by ELISA using SARS-CoV-2 and SARS-CoV-1 Spike S1 recombinant protein, according to the manufacturer and ELISA validation was confirmed in our laboratory using commercially available recombinant SARS-CoV-2 Spike. Goat anti-human IgG (H+L) Alexa647 secondary antibody (Invitrogen, Catalog # A-21445) was validated by relative expression to ensure that it binds to the antigen stated, according to the manufacturer. Human monoclonal antibody 17b was produced in human cells from available sequences and was validated by ELISA using recombinant HIV Envelope protein. CV3 antibodies were produced in human cells from available sequences and validated by ELISA using SARS-CoV-2 recombinant Spike protein (Beaudoin-Bussieres et al., STAR Protocols, 2021).

Eukaryotic cell lines

Policy information about cell lines and Sex and Gender in Research

Cell line source(s)	CEM.NKr-CCR5 direct source was Andres Finzi laboratory (original HIV Reagent Program #4376) and CEM.NKr-CCR5-Spike cells were derived from CEM.NKr-CCR5 cells (Anand et al., 2021b); HEK293T cells used for pseudovirus production were sourced from ATCC; HEK293T-ACE2 cells were a gift from Jesse Bloom at Fred Hutchinson Cancer Center and originally sourced from BEI Resources (NR-52511).
Authentication	CEM.NKr-CCR5 cells were PCR/STR authenticated by HIV Reagent Program.. CEM.NKr-CCR5-Spike cells were PCR/STR authenticated by HIV Reagent Program and confirmed for Spike-GFP expression by flow cytometry and cell-surface staining (Beaudoin-Bussieres, STAR Protocols, 2021).<br>HEK293T cells were PCR/STR authenticated by ATCC. HEK293T-ACE2 cells were PCR/STR authenticated by ATCC and ACE2 expression was confirmed using anti-human ACE2 polyclonal goat IgG (Crawford et al., Viruses, 2020).
Mycoplasma contamination	Cell lines were confirmed negative for mycoplasma upon distribution or during routine mycoplasma testing in our laboratory.
Commonly misidentified lines<br>(See ICLAC register)	No commonly misidentified cell lines were used in this study.

Flow Cytometry

Plots

Confirm that:

- ☒ The axis labels state the marker and fluorochrome used (e.g. CD4-FITC).
- ☒ The axis scales are clearly visible. Include numbers along axes only for bottom left plot of group (a 'group' is an analysis of identical markers).
- ☒ All plots are contour plots with outliers or pseudocolor plots.
- ☒ A numerical value for number of cells or percentage (with statistics) is provided.

Methodology

Sample preparation	Cell surface staining: 300,000 parental or Spike-expressing CEM (S-CEM) cells were stained with plasma (1:500 final dilution) or control mAbs (1 µg/mL final concentration) for 45 min at RT. Cells were washed twice with PBS and 100L of Goat anti-human IgG (H+L) Alexa647 secondary antibody (2 µg/mL, Invitrogen) and Aqua viability dye was added for 20 minutes at RT. After staining, cells were washed twice with PBS and fixed with 2% formaldehyde in PBS.<br><br>ADCC: S-expressing cells were mixed at a 1:1 ratio with parental CEM.NKr CCR5+ cells (HIV Reagent Program #4376) and the target cell mixture was labelled with Aqua viability dye (Thermo Fisher Scientific) and eBioScience eFluor670 cell proliferation dye. In parallel, PBMCs from healthy uninfected adult individuals were labelled with eBioScience eFluor450 cell proliferation dye after overnight rest to use as effectors in the assay. PBMCs from a single donor were used in all replicate experiments. Labelled target and effector cells were added to 96-well V bottom plates at a 1:10 ratio. Plasma (1:500 final dilution) or control monoclonal antibodies (1µg/mL final concentration) were added to corresponding wells and wells were mixed by pipetting up and down. Plates were then centrifuged for 1 min at 300g to bring the cells into close association. ADCC was allowed to occur for 5 hours at 37°C, after which cells were fixed in 2% formaldehyde in PBS.
Instrument	Cell surface staining and ADCC flow cytometry conducted on the LSRII instrument (BD Biosciences).
Software	Standard, built-in LSRII software used for data collection (no custom code). Data analysis was conducted using FlowJo v10.7.1 (TreeStar).
Cell population abundance	A minimum of 10,000 Live eFluor670+ eFluor450- target cell events recorded per sample for ADCC experiments. Likewise, a minimum of 10,000 events were collected for cell surface stained Spike-CEM cells.
Gating strategy	For cell surface staining, cells were gated according to cell morphology by light-scatter parameters and excluding doublets cells. Dead cells (AquaHigh) were then excluded. Finally, the GFP+ cells were used to detect and measure the Spike-specific antibodies present in the plasma.<br><br>For ADCC, target cells were identified according to cell morphology by light-scatter parameters and excluding doublets. Cells were then gated on eFluor-670+ cells (excluding the effector cells labeled with eFluor-450). Finally, the percentage of GFP+ target cells was used to calculate the ADCC activity.

☒ Tick this box to confirm that a figure exemplifying the gating strategy is provided in the Supplementary Information.
